# Japan’s initiative on rare and undiagnosed diseases (IRUD): towards an end to the diagnostic odyssey

**DOI:** 10.1038/ejhg.2017.106

**Published:** 2017-07-05

**Authors:** Takeya Adachi, Kazuo Kawamura, Yoshihiko Furusawa, Yuji Nishizaki, Noriaki Imanishi, Senkei Umehara, Kazuo Izumi, Makoto Suematsu

**Affiliations:** 1 Japan Agency for Medical Research and Development (AMED), Tokyo, Japan

## Abstract

Japan has been facing challenges relating to specifically defined rare diseases, called *Nan-Byo* in Japanese (literally ‘difficult’+‘illness’), and has already taken measures for them since 1972. This governmental support has surely benefited *Nan-Byo* patients; however, those suffering from medically unidentified conditions do not fall into this scheme and thus still confront difficulty in obtaining an examination, a diagnosis, and a treatment. To identify such rare and often undiagnosed diseases, we must integrate systematic diagnosis by medical experts with phenotypic and genetic data matching. Thus, in collaboration with *Nan-Byo* researchers and the Japanese universal healthcare system, the Japan Agency for Medical Research and Development launched the Initiative on Rare and Undiagnosed Diseases (IRUD) in 2015. IRUD is an ambitious challenge to construct a comprehensive medical network and an internationally compatible data-sharing framework. Synergizing with existing next-generation sequencing capabilities and other infrastructure, the nationwide medical research consortium has successfully grown to accept more than 2000 undiagnosed registrants by December 2016. We also aim at expanding the concept of microattribution throughout the initiative; that is, proper credit as collaborators shall be given to local primary care physicians, nurses and paramedics, patients, their family members, and those supporting the affected individuals whenever appropriate. As it shares many challenges among similar global efforts, IRUD’s future successes and lessons learned will significantly contribute to ongoing international endeavors, involving players in basic research, applied research, and societal implementation.

## The mission

**Figure 1:**
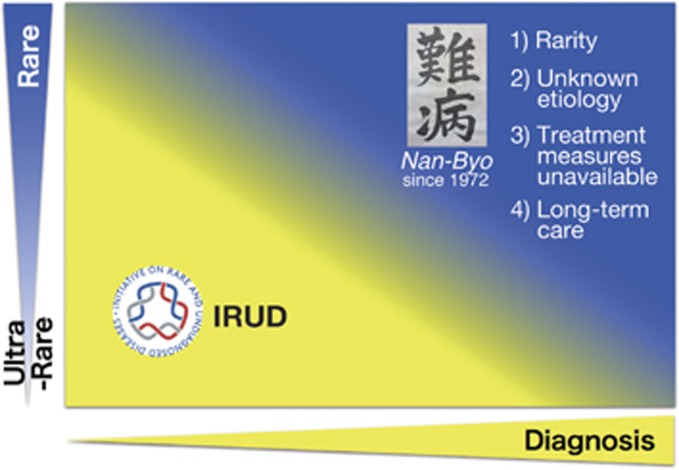
IRUD’s mission within the existing healthcare delivery architecture in Japan. A disease is referred to as *Nan-Byo* if it is a rare disease of unknown etiology which requires currently unavailable treatment measures and long-term care for the patient. Research funding has been available for various *Nan-Byo* projects; and national subsidiary structures have helped offset the cost incurred by patients suffering from eligible *Nan-Byo* diseases, which had received nomination, consultation, and final authorization by MHLW as *designated Nan-Byo*. IRUD is positioned to expand the horizon of *Nan-Byo* research, reaching out to people suffering from unidentified diseases.

Japan has a long research history of pursuing rare and intractable diseases. The Japanese word *Nan-Byo* (literally ‘difficult’+‘illness’) was coined nearly half a century ago when significant but relatively rare health issues occurred in Japan, and the public definition of *Nan-Byo* first appeared in 1972.^1^ Since then the Ministry of Health, Labour and Welfare of Japan (MHLW) has played an essential role in supporting *Nan-Byo* research and subsidizing designated *Nan-Byo* patients to offset their out-of-pocket medical expenses (Figure 1).^2, 3^ As individual diseases were examined against the predetermined definition, the number of designated *Nan-Byo* gradually increased from 4 in 1972 to 56 by early 2014, and then has soared to hundreds—expected to be 330 in April 2017—following legal reform that established national subsidiary structures. The disorders covered included many difficult-to-diagnose diseases, particularly cases in which accurate diagnostic definitions remained undetermined due to the lack of sufficient, well-coordinated, and integrated information.

In the meantime, the use of advanced technology in medical diagnostics has surged over the last few decades. Among other technological developments, next-generation sequencing (NGS) is having a major impact and is drastically improving our ability to find genes associated with Mendelian disorders. Since some 3000 of the estimated total 7000 single-gene disorders have little or no information about the genes responsible for their etiologies, NGS-based, clinically amenable approaches are expected to provide critical clues regarding, for example, whether a certain disease is caused by a previously unknown gene or a known gene whose identification is impeded by the rarity of the disease.

Technology by itself, though, will not address the needs of undiagnosed patients longing for a diagnosis. For example, how does a patient consult a doctor and eventually be referred to an appropriate medical specialist without delay? How can s/he be assessed for further testing leading to a diagnosis? In the case of a rare disease, how can clinical information be securely treated and still participate in patient matching outside of the medical center? How can we take full advantage of this precious knowledge for diagnosing, and hopefully treating future patients? We must be aware of such challenges that underscore the suffering of patients and their families in a long sequence of investigations and referrals, metaphorically speaking, ‘the diagnostic odyssey.’ Indeed, we need strategies to establish a systematic approach, and we have to admit that there are some issues beyond our current capacity. Latter issues include a controversy over the Japanese public health insurance coverage along with reimbursement of genetic services (still limited to 71 applicable diseases in 2016^4^), and significantly fewer clinical geneticists relative to the population size of, for example, the United States. Nevertheless, we also realize that it is the time to tackle these challenges under Japan’s unique, comprehensive approach towards universal care.

The Initiative on Rare and Undiagnosed Diseases (IRUD), led and coordinated by the Japan Agency for Medical Research and Development (AMED), was initiated in 2015 to accelerate the pioneering efforts made by international counterparts. When novel programs for undiagnosed patients (the Undiagnosed Diseases Program/Network (UDP/UDN) in the United States,^5, 6^ the Finding of Rare Disease Genes (FORGE) program in Canada,^7^ and the Deciphering Developmental Disorders (DDD) in the United Kingdom,^8^ to name a few) were launched, Japan had already been developing ‘testbeds’ of NGS-based research for years in various realms, including neuropathy,^9^ myopathy,^10^ pediatrics^11^ and other *Nan-Byo*-related areas. Capitalizing on these existing NGS and other technology cores, and introducing multiple perspectives in a series of clinical conferences, we took another step forward to maximize the benefit of whole-exome and whole-genome analyses for patients searching for a diagnosis.

## The core aims

IRUD has been seeking, and will continue to seek, a growing network consisting of medical experts and the patients themselves. At its core, there exist principal research groups that drive IRUD toward the accomplishment of its goals, but the term ‘experts’ does not limit the participation of non-research sectors. Rather, it is equally important to recognize expanded microattribution by properly giving credit as IRUD collaborators to local primary care physicians, nurses and paramedics, patients, their family members, and those supporting the affected individuals.

**Figure 2:**
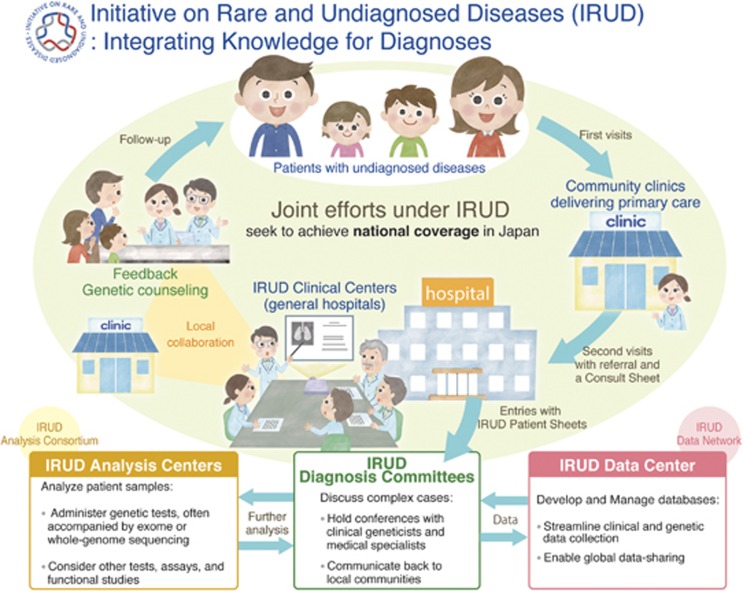
Schematic workflow of the IRUD operation. The three major functional modules—Diagnosis Committees and the local collaboration, Data Network, and Analysis Consortium—closely interact with one another and are operated by principal research groups.

With that in mind, during the first few years significant effort was expended to build up a nationwide medical research consortium dedicated to either pediatric or adult patients (Figure 2). Collaborating with each other, the pediatric and adult research consortia strive to (1) reach out to potential participating institutions and co-ordinate them as the ‘All-Japan’ diagnosis system for those who are currently undiagnosed; (2) develop globally compatible databases and identify data-sharing opportunities; and (3) accelerate research and development in the field of rare and undiagnosed diseases.

Addressing these challenges it is anticipated that, under the name of IRUD, the local-to-central coordination will be seamlessly achieved and sustainably maintained within respective geographic service areas. When accepting first visits of patients, the clinics will use a defined format to collect clinical information, which is practically designed as a ‘consult sheet.’ Thus, attempts to diagnose are made first at local clinics, and in difficult cases the physicians can consider referring each patient to one of IRUD Clinical Centers when the following criteria are met:

1. The patient remains undiagnosed for six months or longer (not necessary for infants) and the symptom(s) affects his/her daily life; AND,

2-1. There exists an objective sign(s) that cannot be reduced to a single organ; OR

2-2. There exists direct or indirect evidence of a genetic etiology as likely (e.g., similar symptom(s) found in the patient’s relatives).

After entry to the IRUD diagnosis scheme, clinical information and other supplementary data are associated and compared with cases stored in the database. This critical step is meant to screen out ‘less rare’ cases that can be referred to existing researchers at, for example, the above-mentioned NGS cores, whereas in return it allows elusive cases from those cores to be entered into the IRUD scheme. Upon completion, feedback is provided to individual patients, preferably by genetic counselors.

To date, AMED has funded some 600–700 million JPY (~6 million USD) annually, materializing a registry of more than 2000 undiagnosed patients by December 2016 and covering the cost of whole-exome sequencing. During the process, the nationwide medical research consortium has grown to involve 34 Clinical Centers, 4 Analysis Centers and 1 Data Center, supported by ~500 physicians and 50 coordinators joining the Diagnosis Committees. Patient registration is only increasing as the IRUD activities become widely disseminated to university hospitals, community clinicians, and municipalities, thanks to partnerships with corresponding liaison committees and associations.

IRUD is an integrative effort to empower existing medical infrastructure. We acknowledge that the majority of registered patients remain undiagnosed even after mutual referral within the cluster of related research consortia; however, intensive research on specific cases, accompanied by training for nationwide collaborators by the IRUD network, will potentially lead to diagnostic and therapeutic innovations in both the short- and long-term. For these goals to actualize, the Japanese long research history and well-maintained population data^12^ must, and will, be revisited under the IRUD umbrella. This ambition distinguishes IRUD from any simple referral program, leaving room for evolution beyond diagnostic improvement as we recently set forth as part of its future directions.^13^

## The global impacts

As many challenges are shared between IRUD and similar global efforts, collaboration and harmonization will be another important key to success.^13^ For example, once registered, patients’ clinical information must be securely managed but adequately shared to improve diagnostic discovery. Ideally, it comes with such metadata as permissions, limitations and conditions of use in discovery and access scenarios, which has been formulated by the International Rare Diseases Research Consortium (IRDiRC) as the Automatable Discovery and Access-Matrix.^14^ We recognize the importance of such global approaches to common solutions; thus, IRUD will try to become another pioneering testbed that adheres to guidelines recommended by international forums.

IRUD and related national projects—which means AMED as a whole—maintain close ties to MHLW with respect to implementation in public health. Collective progress will ensure that a wide range of experts and institutions will be optimally positioned in the Japanese universal healthcare system, enhancing IRUD’s potential capability to search for ‘hidden jewels’ that might otherwise be inaccessible and thus overlooked. As new discoveries are delivered by the greater *Nan-Byo* community, IRUD will foster their translation, ultimately contributing to national engagement promoted by the Japanese government.^15^

IRUD played a founding role in UDN International,^6^ and similarly, IRUD’s future successes and lessons learned will be linked to our pioneering activities in additional international endeavors. IRDiRC, a consortium to accelerate rare disease research through international collaborations, will work to achieve its global objectives by 2027 [tentative]: diagnosis for all patients having an identified rare disease, or enabling all currently undiagnosable cases to enter a globally coordinated diagnostic and research pipeline.^16^ Toward this futuristic global goal, AMED joins forces with IRUD and many other organizations to realize a new world for undiagnosed patients with rare diseases.
